# *N*-(Diethyl­carbamothio­yl)-4-nitro­benzamide

**DOI:** 10.1107/S1600536810036366

**Published:** 2010-09-18

**Authors:** Sohail Saeed, Naghmana Rashid, Jerry P. Jasinski, Ray J. Butcher, Hussain Rizwan

**Affiliations:** a Department of Chemistry, Research Complex, Allama Iqbal Open University, Islamabad, Pakistan; b Department of Chemistry, Keene State College, 229 Main Street, Keene, NH 03435-2001, USA; c Department of Chemistry, Howard University, 525 College Street NW, Washington, DC 20059, USA; d National Engineering & Scientific Commission, PO Box 2801, Islamabad, Pakistan

## Abstract

In the title compound, C_12_H_15_N_3_O_3_S, the 4-nitro and carbonyl groups are nearly coplanar with the benzene ring [C—C—N—O = −175.72 (14) and C—C—C—O = 172.75 (14)°]. The diethyl­carbamothioyl group is twisted significantly from the plane of the benzene ring [C—N—C—N = −89.79 (15)°] with the S atom pointing away from each of these groups [C—N—C—S = 91.12 (14)°]. In the crystal, an inter­molecular N—H⋯O hydrogen bond, which forms an infinite polymeric chain along the *c* axis, and weak C—H⋯O and C—H⋯S hydrogen bonds are observed.

## Related literature

For background to the use of thio­ureas in coordination chemistry, see: Burrows *et al.* (1999 ▶); Henderson *et al.* (2002 ▶), Schuster *et al.* (1990 ▶); Che *et al.* (1999 ▶); For their biological and catalytic activity, see: Saeed *et al.* (2009 ▶, 2010*a* ▶,*b* ▶); Maddani *et al.* (2010 ▶); Jung *et al.* (2008 ▶); For related literature, see: Zhang *et al.* (2004 ▶). For bond-length data, see: Allen *et al.* (1987 ▶).
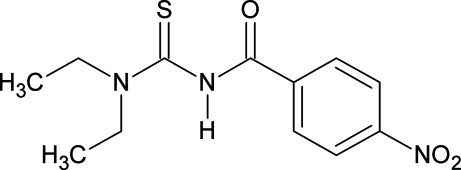


## Experimental

### 

#### Crystal data

C_12_H_15_N_3_O_3_S
*M* *_r_* = 281.33
Monoclinic, 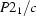

*a* = 6.884 (5) Å
*b* = 19.237 (5) Å
*c* = 10.146 (5) Å
β = 92.983 (5)°
*V* = 1341.8 (12) Å^3^
*Z* = 4
Cu *K*α radiation
μ = 2.23 mm^−1^
*T* = 295 K
0.52 × 0.41 × 0.35 mm

#### Data collection

Oxford Diffraction Xcalibur Ruby Gemini Cu diffractometer
Absorption correction: multi-scan (*CrysAlis RED*; Oxford Diffraction, 2007 ▶) *T*_min_ = 0.769, *T*_max_ = 1.000
5616 measured reflections
2790 independent reflections
2507 reflections with *I* > 2σ(*I*)
*R*_int_ = 0.018

#### Refinement

*R*[*F* ^2^ > 2σ(*F* ^2^)] = 0.040
*wR*(*F* ^2^) = 0.115
*S* = 1.05
2790 reflections
178 parameters
H atoms treated by a mixture of independent and constrained refinement
Δρ_max_ = 0.25 e Å^−3^
Δρ_min_ = −0.30 e Å^−3^

### 

Data collection: *CrysAlis PRO* (Oxford Diffraction, 2007 ▶); cell refinement: *CrysAlis RED*; data reduction: *CrysAlis RED*; program(s) used to solve structure: *SHELXS97* (Sheldrick, 2008 ▶); program(s) used to refine structure: *SHELXL97* (Sheldrick, 2008 ▶); molecular graphics: *SHELXTL* (Sheldrick, 2008 ▶); software used to prepare material for publication: *PLATON* (Spek, 2009 ▶).

## Supplementary Material

Crystal structure: contains datablocks global, I. DOI: 10.1107/S1600536810036366/vm2042sup1.cif

Structure factors: contains datablocks I. DOI: 10.1107/S1600536810036366/vm2042Isup2.hkl

Additional supplementary materials:  crystallographic information; 3D view; checkCIF report

## Figures and Tables

**Table 1:** Hydrogen-bond geometry (Å, °)

*D*—H⋯*A*	*D*—H	H⋯*A*	*D*⋯*A*	*D*—H⋯*A*
N2—H2*B*⋯O3^i^	0.85 (2)	2.08 (2)	2.915 (2)	165.4 (19)
C2—H2*A*⋯O3^i^	0.93	2.56	3.4671 (19)	165
C6—H6*A*⋯S1^ii^	0.93	2.98	3.824 (2)	152

Symmetry codes: (i) 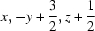; (ii) 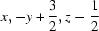.

**Fig. 1.**
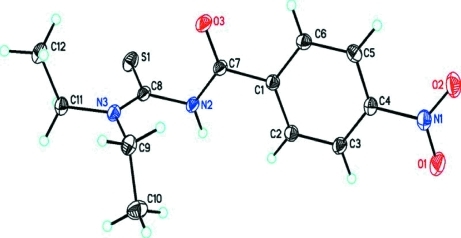
Molecular structure of C12H15N3O3S showing the atom labeling scheme and 50% probability displacement ellipsoids.

**Fig. 2.**
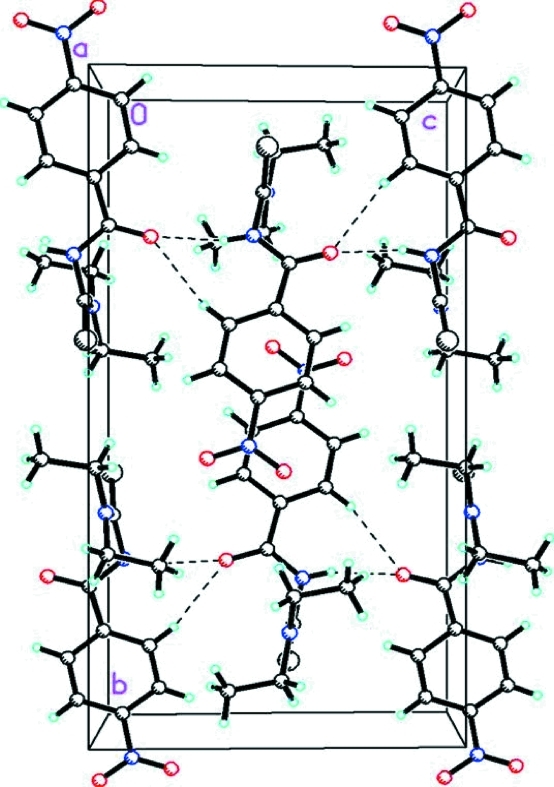
Packing diagram of the title compound C12H15N3O3S viewed down the a axis. Dashed lines indicate N—H···O and weak C—H···O hydrogen bonds.

## supplementary crystallographic information

### Comment

Thioureas are of significant interest in medicinal chemistry due to their biological activity as fungicides (Saeed *et al.*, 2010*a*), anticancer (Saeed *et al.*, 2010*b*), herbicides, rodenticides and phenoloxidase enzymatic inhibitors (Maddani *et al.*, 2010). Recently, thiourea derivatives have found use in organocatalysis (Jung *et al.*, 2008) and amino-thiourea derivatives as curing agents for epoxy resins (Saeed *et al.*, 2009). Thioureas have a long history as ligand in coordination chemistry and coordinate to a metal *via* sulfur (Burrows *et al.*, 1999). These hard and soft donor atoms provide a multitude of bonding possibilities (Henderson *et al.*, 2002). Hydrogen bonding behavior of some acyl thioureas has been investigated and it is found that intramolecular hydrogen bonds between the carbonyl oxygen and a hydrogen atom on N' is common. The complexing capacity of thiourea derivatives has been reported in several papers (Schuster *et al.*, 1990). Some acyl thioureas possess pesticidal activities and promote plant growth while some have a notable positive effect on the germination of maize seeds as well as on the chlorophyll contents in seedling leaves (Che *et al.*, 1999). With the simultaneous presence of S, N and O electron donors, the versalitility and interesting behavior of acyl thioureas as building blocks in polydentate ligands for metal ions have become a topic of interest in the last few years. It has been reported that substituted acyl thiourea ligands might act as monodentate sulfur donors, bidentate oxygen and nitrogen donors. In continuation of our research on the structural modification of certain biologically active thiourea derivatives and their transition metal complexes with the purpose of enhancing their biological activity, we aimed to incorporate aliphatic and aromatic moieties in the substituted phenyl nucleus with thiourea functionality to obtain new functions with improved antimicrobial profile. In view of the importance of thiourea derivatives, the crystal structure of the title compound is reported.

In the title compound, C_12_H_15_N_3_O_3_S the 4-nitro (C3/C4/N1/O2 = -175.72 (14)°) and carbonyl (C2/C1/C7/O3 = 172.75 (14)°) groups are nearly co-planar to the benzene ring. The *N*-(diethylcarbamothioyl) group is significantly twisted from the plane of the benzene ring (C7/N2/C8/N3 = -89.79 (15)°) with the S1 atom pointing away from each of these moieties (C7/N2/C8/S1 = 91.12 (14)°). Bond distances (Allen *et al.*, 1987) and angles are in normal ranges. An N—H···O hydrogen bond which forms an infinite polymeric chain along the *c* axis and weak C—H···O and C—H···S hydrogen bond intermolecular interactions are observed (Table 1) and contribute to crystal packing (Fig. 1).

### Experimental

A solution of 4-nitrobenzoyl chloride (0.01 mol) in anhydrous acetone (80 ml) and 3% tetrabutylammonium bromide (TBAB) as a phase-transfer catalyst (PTC) in anhydrous acetone was added dropwise to a suspension of dry ammonium thiocyanate (0.01 mol) in acetone (50 ml) and the reaction mixture was refluxed for 45 min. After cooling to room temperature, a solution of diethylamine (0.01 mol) in anhydrous acetone (25 ml) was added dropwise and the resulting mixture refluxed for 2.5 h. Hydrochloric acid (0.1 N, 300 ml) was added, and the solution was filtered. The solid product was washed with water and purified by re-crystallization from ethyl acetate.

### Refinement

The N–H atom length was set to 0.85Å (NH) and the H atom refined isotropically. All of the other H atoms were placed in their calculated positions and then refined using the riding model with Atom–H lengths of 0.93Å (CH), 0.97Å (CH_2_), or 0.97Å (CH_3_). Isotropic displacement parameters for these atoms were set to 1.18–1.20 (CH), 1.20 (CH_2_) or 1.50 (CH_3_)times *U*_eq_ of the parent atom.

### Crystal data

**Table:**

C_12_H_15_N_3_O_3_S	*F*(000) = 592
*M**_r_* = 281.33	*D*_x_ = 1.393 Mg m^−^^3^
Monoclinic, *P*2_1_/*c*	Melting point: 434 K
Hall symbol: -P 2ybc	Cu *K*α radiation, λ = 1.54178 Å
*a* = 6.884 (5) Å	Cell parameters from 4133 reflections
*b* = 19.237 (5) Å	θ = 4.6–77.2°
*c* = 10.146 (5) Å	µ = 2.23 mm^−^^1^
β = 92.983 (5)°	*T* = 295 K
*V* = 1341.8 (12) Å^3^	Block, pale yellow
*Z* = 4	0.52 × 0.41 × 0.35 mm

### Data collection

**Table:**

Oxford Diffraction Xcalibur Ruby Gemini Cu diffractometer	2790 independent reflections
Radiation source: Enhance (Cu) X-ray Source	2507 reflections with *I* > 2σ(*I*)
graphite	*R*_int_ = 0.018
Detector resolution: 10.5081 pixels mm^-1^	θ_max_ = 77.4°, θ_min_ = 4.9°
ω scans	*h* = −8→8
Absorption correction: multi-scan (*CrysAlis RED*; Oxford Diffraction, 2007)	*k* = −17→24
*T*_min_ = 0.769, *T*_max_ = 1.000	*l* = −12→12
5616 measured reflections	

### Refinement

**Table:**

Refinement on *F*^2^	Primary atom site location: structure-invariant direct methods
Least-squares matrix: full	Secondary atom site location: difference Fourier map
*R*[*F*^2^ > 2σ(*F*^2^)] = 0.040	Hydrogen site location: inferred from neighbouring sites
*wR*(*F*^2^) = 0.115	H atoms treated by a mixture of independent and constrained refinement
*S* = 1.05	*w* = 1/[σ^2^(*F*_o_^2^) + (0.078*P*)^2^ + 0.1589*P*] where *P* = (*F*_o_^2^ + 2*F*_c_^2^)/3
2790 reflections	(Δ/σ)_max_ < 0.001
178 parameters	Δρ_max_ = 0.25 e Å^−^^3^
0 restraints	Δρ_min_ = −0.29 e Å^−^^3^

### Special details

**Table:**

Geometry. All e.s.d.'s (except the e.s.d. in the dihedral angle between two l.s. planes) are estimated using the full covariance matrix. The cell e.s.d.'s are taken into account individually in the estimation of e.s.d.'s in distances, angles and torsion angles; correlations between e.s.d.'s in cell parameters are only used when they are defined by crystal symmetry. An approximate (isotropic) treatment of cell e.s.d.'s is used for estimating e.s.d.'s involving l.s. planes.
Refinement. Refinement of *F*^2^ against ALL reflections. The weighted *R*-factor *wR* and goodness of fit *S* are based on *F*^2^, conventional *R*-factors *R* are based on *F*, with *F* set to zero for negative *F*^2^. The threshold expression of *F*^2^ > σ(*F*^2^) is used only for calculating *R*-factors(gt) *etc*. and is not relevant to the choice of reflections for refinement. *R*-factors based on *F*^2^ are statistically about twice as large as those based on *F*, and *R*- factors based on ALL data will be even larger.

### Fractional atomic coordinates and isotropic or equivalent isotropic displacement parameters (Å^2^)

**Table:**

	*x*	*y*	*z*	*U*_iso_*/*U*_eq_	
S1	0.38042 (6)	0.89794 (2)	0.52976 (4)	0.05321 (16)	
O1	0.2051 (3)	0.42290 (7)	0.69201 (14)	0.0818 (5)	
O2	0.1805 (2)	0.40370 (6)	0.48459 (14)	0.0627 (3)	
O3	0.4507 (2)	0.73790 (6)	0.35424 (9)	0.0547 (3)	
N1	0.21185 (19)	0.44183 (6)	0.57929 (14)	0.0461 (3)	
N2	0.48161 (17)	0.76526 (6)	0.56885 (10)	0.0364 (3)	
H2B	0.465 (3)	0.7568 (10)	0.650 (2)	0.054 (5)*	
N3	0.73455 (16)	0.84093 (5)	0.53139 (10)	0.0350 (2)	
C1	0.36465 (18)	0.65019 (7)	0.50551 (12)	0.0337 (3)	
C2	0.3617 (2)	0.62603 (7)	0.63527 (12)	0.0387 (3)	
H2A	0.3946	0.6558	0.7051	0.046*	
C3	0.3098 (2)	0.55777 (7)	0.66026 (13)	0.0404 (3)	
H3A	0.3079	0.5412	0.7463	0.048*	
C4	0.26114 (19)	0.51499 (7)	0.55432 (13)	0.0366 (3)	
C5	0.2607 (2)	0.53744 (7)	0.42543 (13)	0.0417 (3)	
H5A	0.2265	0.5075	0.3561	0.050*	
C6	0.3125 (2)	0.60570 (7)	0.40138 (13)	0.0398 (3)	
H6A	0.3124	0.6219	0.3150	0.048*	
C7	0.43282 (19)	0.72156 (7)	0.46945 (12)	0.0358 (3)	
C8	0.54477 (19)	0.83458 (6)	0.54204 (11)	0.0343 (3)	
C9	0.8723 (2)	0.78196 (7)	0.54253 (14)	0.0419 (3)	
H9A	0.9859	0.7928	0.4937	0.050*	
H9B	0.8111	0.7411	0.5029	0.050*	
C10	0.9359 (3)	0.76598 (10)	0.68374 (18)	0.0599 (4)	
H10A	1.0291	0.7288	0.6858	0.090*	
H10B	0.8251	0.7523	0.7312	0.090*	
H10C	0.9941	0.8066	0.7241	0.090*	
C11	0.8218 (2)	0.90929 (7)	0.50932 (14)	0.0424 (3)	
H11A	0.9462	0.9121	0.5585	0.051*	
H11B	0.7380	0.9451	0.5423	0.051*	
C12	0.8516 (3)	0.92242 (10)	0.36548 (17)	0.0626 (5)	
H12A	0.9112	0.9671	0.3555	0.094*	
H12B	0.7282	0.9215	0.3169	0.094*	
H12C	0.9343	0.8870	0.3324	0.094*	

### Atomic displacement parameters (Å^2^)

**Table:**

	*U*^11^	*U*^22^	*U*^33^	*U*^12^	*U*^13^	*U*^23^
S1	0.0504 (2)	0.0454 (2)	0.0645 (3)	0.01207 (15)	0.00856 (18)	0.01556 (17)
O1	0.1340 (14)	0.0535 (7)	0.0571 (8)	−0.0315 (8)	−0.0022 (8)	0.0155 (6)
O2	0.0811 (9)	0.0375 (6)	0.0697 (8)	−0.0083 (5)	0.0065 (6)	−0.0115 (5)
O3	0.0924 (9)	0.0461 (6)	0.0260 (5)	−0.0114 (5)	0.0046 (5)	0.0040 (4)
N1	0.0475 (6)	0.0360 (6)	0.0549 (7)	−0.0035 (5)	0.0026 (5)	0.0004 (5)
N2	0.0496 (6)	0.0339 (5)	0.0258 (5)	−0.0056 (4)	0.0048 (4)	0.0039 (4)
N3	0.0428 (6)	0.0290 (5)	0.0333 (5)	−0.0008 (4)	0.0032 (4)	0.0024 (4)
C1	0.0386 (6)	0.0338 (6)	0.0288 (6)	0.0001 (5)	0.0030 (4)	−0.0007 (5)
C2	0.0521 (7)	0.0360 (6)	0.0282 (6)	−0.0042 (5)	0.0035 (5)	−0.0018 (5)
C3	0.0515 (7)	0.0386 (7)	0.0313 (6)	−0.0024 (5)	0.0050 (5)	0.0034 (5)
C4	0.0364 (6)	0.0324 (6)	0.0413 (6)	−0.0001 (5)	0.0043 (5)	−0.0002 (5)
C5	0.0494 (7)	0.0400 (7)	0.0354 (6)	−0.0023 (6)	0.0004 (5)	−0.0084 (5)
C6	0.0510 (7)	0.0406 (7)	0.0277 (6)	−0.0011 (5)	0.0007 (5)	−0.0010 (5)
C7	0.0442 (6)	0.0351 (6)	0.0282 (6)	0.0001 (5)	0.0033 (5)	0.0035 (5)
C8	0.0460 (7)	0.0311 (6)	0.0259 (5)	−0.0003 (5)	0.0034 (5)	0.0032 (4)
C9	0.0457 (7)	0.0342 (6)	0.0463 (7)	0.0043 (5)	0.0085 (6)	0.0004 (5)
C10	0.0665 (10)	0.0546 (9)	0.0576 (10)	0.0181 (8)	−0.0072 (8)	0.0076 (8)
C11	0.0503 (8)	0.0336 (6)	0.0433 (7)	−0.0077 (5)	0.0013 (6)	0.0024 (5)
C12	0.0816 (12)	0.0578 (10)	0.0486 (9)	−0.0237 (9)	0.0034 (8)	0.0158 (7)

### Geometric parameters (Å, °)

**Table:**

S1—C8	1.6632 (14)	C3—H3A	0.9300
O1—N1	1.203 (2)	C4—C5	1.377 (2)
O2—N1	1.2191 (19)	C5—C6	1.386 (2)
O3—C7	1.2228 (17)	C5—H5A	0.9300
N1—C4	1.4727 (17)	C6—H6A	0.9300
N2—C7	1.3423 (17)	C9—C10	1.508 (2)
N2—C8	1.4331 (16)	C9—H9A	0.9700
N2—H2B	0.85 (2)	C9—H9B	0.9700
N3—C8	1.322 (2)	C10—H10A	0.9600
N3—C11	1.4678 (17)	C10—H10B	0.9600
N3—C9	1.4792 (17)	C10—H10C	0.9600
C1—C6	1.3921 (18)	C11—C12	1.506 (2)
C1—C2	1.3973 (18)	C11—H11A	0.9700
C1—C7	1.5022 (18)	C11—H11B	0.9700
C2—C3	1.3877 (19)	C12—H12A	0.9600
C2—H2A	0.9300	C12—H12B	0.9600
C3—C4	1.3810 (19)	C12—H12C	0.9600
			
O1—N1—O2	123.58 (14)	N2—C7—C1	117.30 (11)
O1—N1—C4	118.24 (13)	N3—C8—N2	114.35 (11)
O2—N1—C4	118.18 (13)	N3—C8—S1	126.68 (10)
C7—N2—C8	120.45 (10)	N2—C8—S1	118.96 (10)
C7—N2—H2B	124.5 (14)	N3—C9—C10	112.48 (12)
C8—N2—H2B	114.6 (14)	N3—C9—H9A	109.1
C8—N3—C11	120.62 (11)	C10—C9—H9A	109.1
C8—N3—C9	123.73 (11)	N3—C9—H9B	109.1
C11—N3—C9	115.64 (12)	C10—C9—H9B	109.1
C6—C1—C2	119.65 (13)	H9A—C9—H9B	107.8
C6—C1—C7	116.65 (12)	C9—C10—H10A	109.5
C2—C1—C7	123.60 (11)	C9—C10—H10B	109.5
C3—C2—C1	120.22 (12)	H10A—C10—H10B	109.5
C3—C2—H2A	119.9	C9—C10—H10C	109.5
C1—C2—H2A	119.9	H10A—C10—H10C	109.5
C4—C3—C2	118.43 (12)	H10B—C10—H10C	109.5
C4—C3—H3A	120.8	N3—C11—C12	112.06 (12)
C2—C3—H3A	120.8	N3—C11—H11A	109.2
C5—C4—C3	122.73 (13)	C12—C11—H11A	109.2
C5—C4—N1	118.29 (12)	N3—C11—H11B	109.2
C3—C4—N1	118.97 (12)	C12—C11—H11B	109.2
C4—C5—C6	118.46 (12)	H11A—C11—H11B	107.9
C4—C5—H5A	120.8	C11—C12—H12A	109.5
C6—C5—H5A	120.8	C11—C12—H12B	109.5
C5—C6—C1	120.50 (12)	H12A—C12—H12B	109.5
C5—C6—H6A	119.8	C11—C12—H12C	109.5
C1—C6—H6A	119.8	H12A—C12—H12C	109.5
O3—C7—N2	121.57 (13)	H12B—C12—H12C	109.5
O3—C7—C1	121.07 (12)		
			
C6—C1—C2—C3	0.9 (2)	C8—N2—C7—C1	−178.94 (11)
C7—C1—C2—C3	−175.39 (13)	C6—C1—C7—O3	−3.7 (2)
C1—C2—C3—C4	−0.2 (2)	C2—C1—C7—O3	172.75 (14)
C2—C3—C4—C5	−0.5 (2)	C6—C1—C7—N2	179.30 (12)
C2—C3—C4—N1	178.08 (12)	C2—C1—C7—N2	−4.3 (2)
O1—N1—C4—C5	−177.27 (16)	C11—N3—C8—N2	−177.53 (11)
O2—N1—C4—C5	2.9 (2)	C9—N3—C8—N2	1.45 (17)
O1—N1—C4—C3	4.1 (2)	C11—N3—C8—S1	1.47 (17)
O2—N1—C4—C3	−175.72 (14)	C9—N3—C8—S1	−179.55 (10)
C3—C4—C5—C6	0.4 (2)	C7—N2—C8—N3	−89.79 (15)
N1—C4—C5—C6	−178.16 (13)	C7—N2—C8—S1	91.12 (14)
C4—C5—C6—C1	0.4 (2)	C8—N3—C9—C10	−84.81 (17)
C2—C1—C6—C5	−1.0 (2)	C11—N3—C9—C10	94.21 (15)
C7—C1—C6—C5	175.56 (13)	C8—N3—C11—C12	−96.35 (16)
C8—N2—C7—O3	4.1 (2)	C9—N3—C11—C12	84.59 (16)

### Hydrogen-bond geometry (Å, °)

**Table:**

*D*—H···*A*	*D*—H	H···*A*	*D*···*A*	*D*—H···*A*
N2—H2B···O3^i^	0.85 (2)	2.08 (2)	2.915 (2)	165.4 (19)
C2—H2A···O3^i^	0.93	2.56	3.4671 (19)	165
C6—H6A···S1^ii^	0.93	2.98	3.824 (2)	152

Symmetry codes: (i) *x*, −*y*+3/2, *z*+1/2; (ii) *x*, −*y*+3/2, *z*−1/2.
